# Enhanced Recovery After Surgery Protocol Optimizes Results and Cost of Laparoscopic Radical Nephrectomy

**DOI:** 10.3389/fonc.2022.840363

**Published:** 2022-04-04

**Authors:** Siming Chen, Zhiwen He, Shijie Yao, Kangping Xiong, Jiageng Shi, Gang Wang, Kaiyu Qian, Xinghuan Wang

**Affiliations:** ^1^ Department of Urology, Zhongnan Hospital of Wuhan University, Wuhan, China; ^2^ Department of Gynecological Oncology, Zhongnan Hospital of Wuhan University, Wuhan, China; ^3^ Department of Biological Repositories, Zhongnan Hospital of Wuhan University, Wuhan, China; ^4^ Laboratory of Precision Medicine, Zhongnan Hospital of Wuhan University, Wuhan, China; ^5^ Wuhan Research Center for Infectious Diseases and Cancer, Chinese Academy of Medical Sciences, Wuhan, China; ^6^ Department of Biological Repositories, Frontier Science Center for Immunology and Metabolism, Medical Research Institute, Zhongnan Hospital of Wuhan University, Wuhan University, Wuhan, China

**Keywords:** enhanced recovery after surgery, renal cell carcinoma, laparoscopic radical nephrectomy, length of hospital stay, postoperative complications

## Abstract

**Purpose:**

To assess the impact of enhanced recovery after surgery (ERAS) protocols in laparoscopic radical nephrectomy (LRN).

**Methods:**

The clinical data of 89 patients underwent LRN in Zhongnan Hospital of Wuhan University from February 2019 to September 2021 were collected (40 in the ERAS group and 49 in the pre-ERAS group). The clinical characteristics, prognosis, and length of hospital stay (LOS) were compared between the two groups using t test, Mann-Whitney test, and chi-square test.

**Results:**

Total LOS and postoperative LOS were significantly shorter in ERAS group than in pre-ERAS group [15.0 (13.5-19.5) *vs*. 12.0 (10.0-14.0), P < 0.001; 8.0 (7.0-10.0) *vs*. 7.0 (5.0-8.8), P = 0.001]. Compared with the pre-ERAS group, the hospitalization expenses of the ERAS group were also lower (P = 0.023). In addition, the incidence of postoperative complications in the ERAS group also decreased (P = 0.054).

**Conclusions:**

ERAS protocol in LRN could help accelerate the recovery of patients and is worthy of clinical promotion.

## Introduction

As a common malignant tumor of the urinary system, renal cell carcinoma (RCC) seriously endangers people’s health (1, 2). RCC affects more than 430,000 people worldwide annually, and its incidence is increasing by about 2% annually, making it the third most common malignancy of the genitourinary system (3, 4). Clear cell renal cell carcinoma (ccRCC) is the most common form of RCC, accounting for 70-80% of RCC cases. Poor prognosis is typical for RCC due to its high rate of metastasis and difficulty in diagnosis. Smoking, obesity, hypertension and chronic kidney disease are also major risk factors for RCC (1). For patients with localized RCC, surgery is the first treatment of choice (5). Radical nephrectomy (RN) has long been considered the standard treatment for local RCC (6). With the development of minimally invasive surgical techniques, laparoscopic radical nephrectomy (LRN) has become the mainstream surgical method of RN due to its advantages of fewer perioperative complications and quick recovery (7–9). Despite recent evidence showed that partial nephrectomy (PN) is equally suitable in treating T1a (≤ 4 cm) renal tumors, and can better protect renal function (5, 10). However, LRN is still an important treatment for large renal tumors or tumors that are not suitable for nephron-sparing surgery (11).

The concept of enhanced recovery after surgery (ERAS) was first proposed by Professor Kehlet of Denmark and successfully applied to colorectal surgery for the first time (12, 13). It is an emerging surgical protocol that organically integrates anesthesiology, nursing and surgery, and collaborates with multiple disciplines to reduce surgical stress response and complications, and to achieve rapid recovery (14). ERAS is an integrated innovation of perioperative surgical treatment concepts and techniques, which are characterized by: (1) Combined multidisciplinary advantages. (2) Focus on perioperative management. (3) Make use of the charm of new technologies. The advent of minimally invasive era, especially the wide application of minimally invasive techniques such as surgical robots and endoscopes. (4) Optimize clinical pathway and create system evaluation system. (5) Emphasize safe rehabilitation, which is the premise of rapid rehabilitation. Through multi-department and multidisciplinary collaboration in the hospital, our team discussed and summarized ERAS protocols, and applied ERAS protocols to clinical practice, including perioperative education, intestinal management, fluid management, early extubation and activities. ERAS protocol implementation could be divided into three stages: preoperative, intraoperative and postoperative. Individualized treatment is provided according to patient heterogeneity, with emphasis on reduced preoperative fasting time, multi-mode analgesia, early enteral feeding, and early ambulation (15). In the context of the modern medical model, ERAS protocol developed rapidly and has been widely accepted and implemented in various surgical specialties such as neurosurgery, urology and hepatobiliary and pancreatic surgery, significantly reducing the length of hospital stay, medical costs and postoperative complications (16–19). Although ERAS has achieved satisfactory results in other urological procedures, evidence on LRN is sparse and lacking.

Here, we conducted a retrospective study of LRN to analyze the length of hospital stay (LOS), hospitalization costs, and postoperative complications before and after the implementation of the ERAS protocol. The aim of this study was to compare LOS and short-term outcomes after LRN between ERAS patients and conventional care patients, and to provide reference value for future medical decisions.

## Materials and Methods

### Patient Cohort

We retrospectively analyzed RCC patients who underwent LRN in our department before the implementation of ERAS (from Feb. 1^st^, 2019, to Aug. 31^st^, 2020) and after (from Sept. 1^st^, 2020, to Sept. 30^th^, 2021). According to the inclusion and exclusion criteria, a total of 89 patients who received LRN for RCC were included, of which 49 were in the pre-ERAS group and 40 were in the ERAS group. The inclusion criteria were as follows: (1) Clinical diagnosis of primary RCC; (2) Underwent laparoscopic radical nephrectomy; (3) No history of other malignant tumors; (4) > 18 years old. Exclusion criteria: (1) Postoperative pathology indicated non-RCC; (2) Metastatic RCC or other combined tumors; (3) No surgical treatment; (4) No complete clinical information. This study was approved by the Ethics Committee of Zhongnan Hospital (approval number: 2020102), and the informed consent of all participants was obtained.

### Laparoscopic Surgery

All the patients underwent laparoscopic radical nephrectomy *via* retroperitoneal approach. All patients received combined treatment of inhalation and intravenous anesthesia, and ventilation was maintained by ventilator. Laparoscopic surgery was performed by the same group of senior surgeons, with assistants helping to maneuver the endoscope. The patient was placed in the decubitus position, and the same technique was used to place the trocar and insert the laparoscope and operating instruments. The trocars were placed below the 12th costal margin on the posterior axillary line (point A, 10 mm), 1.0 cm above the iliac crest on the mid-axillary line (point B, 10 mm), and below the costal margin on the anterior axillary line (point C, 5 mm). Points A and C were the surgical holes for the surgeon, and point B was used to insert the light source and camera system. After the artificial pneumoperitoneum was established, the kidney was dissociated under the endoscope, and the renal artery and vein were fully exposed and severed, and then the ureter was cut off. The kidneys were removed after complete dissociation, electrocoagulation was performed to completely stop the bleeding, and a drainage tube was placed.

### Pre-ERAS Management

The patients were managed according to the traditional routine mode, preoperative fasting for 12 h, preoperative water deprivation for 4 h, and had a clean enema before surgery. During the surgery, patients were treated with liberal fluid therapy, and vital signs such as blood pressure, respiration, and heart rate were monitored. Strictly fasting and water-free after the operation, and give 2,500-3,000ml fluid daily for 2-3 days. Liquid food could be used after the anus exhaust. Most activities begin after surgery for pain relief, and bedside activities begin on postoperative day (POD) 2 or 3. The urinary tube was removed on POD3 or 4 without special discomfort, and the drainage tube was removed when the postoperative drainage volume was less than 20mL for 2 consecutive days.

### ERAS Management

The responsible nurse, the doctor in charge, the patient and the patient’s family formulated reasonable nursing goals and effective nursing measures according to the patient’s condition. The doctor conducted medical education for the patient and fully communicated with the patient to relieve the patient’s preoperative anxiety. No need for cleaning enema before surgery. Shorten the time of fasting and drinking before surgery. During the operation, preheated intravenous infusion or thermal insulation blanket was used to maintain body temperature. Control intraoperative and postoperative fluid intake. Drink water after the postoperative anesthesia recovery, and liquid food can be taken on POD 1. The patient could get out of bed without special discomfort on POD1. The urinary catheter was removed on POD1 or 2, and the drainage tube was removed when the daily drainage flow was less than 20 ml.

**Table 1** listed the main differences between the pre-ERAS and ERAS protocols.

**Table 1:** Enhanced recovery protocol for laparoscopic radical nephrectomy.

Managements		Pre-ERAS	ERAS
Bowel management	Bowel preparation	Clean the enema before surgery	No enema
	Preoperative fasting (h)	12	6
	Preoperative water deprivation (h)	4	2
	Postoperative fasting (h)	Bowel movement recovered	6 after surgery
	Postoperative water deprivation (h)	POD1	Awakening from anesthesia
Fluid management	Intraoperative intravenous fluid intake	Routine 1500mL	Controlled within 500mL
	Postoperative intravenous fluid intake	Complete intravenous fluids on POD1; Gradually decreases	No more than 1000ml on POD1
Intraoperative management	Intraoperative warming	Not stressed	Preheat intravenous transfusion fluids or warm blankets
Postoperative management	Antibiotic	Routine use of antibiotics	No, rely on the infectious markers
	Mobilization (d)	Sit on the POD1; Walk on the POD2	Sit and walk on the POD1
	Urinary catheter removal (d)	POD3- POD4	POD1- POD2
	Drainage tube removal (d)	Drainage <20ml Observe for another day	Drainage<20ml
Nurse		Mainly focus on postoperative care operations	Pay attention to perioperative care: pre-operative preaching; post-operative treatment will be changed to comprehensive psychological and medical care

ERAS, enhanced recovery after surgery; POD, postoperative day.

### Data Collection, Definitions, and Primary Outcomes

In addition to basic patient information, surgical information, and pathological information, we also collected and analyzed the removal time of urinary catheters and drainage catheters and the corresponding early postoperative complications during hospitalization. Early postoperative complications include urinary retention, venous thrombosis, gastrointestinal discomfort, infection, and postoperative hypotension. Length of hospital stay (LOS) includes total hospital stay and postoperative hospital stay, which are respectively defined as the time from admission to discharge and the time from operation to discharge recorded in the electronic medical record. The patient’s hospitalization expenses include laboratory examination, imaging examination, surgery, medicine, nursing and other expenses. The fasting peripheral venous blood was collected before surgery and on POD1 to detect the renal function indicators (blood urea nitrogen and creatinine) before and after surgery. LOS and hospitalization expenses were considered as primary outcomes, while the incidence of postoperative complications and catheter and drainage removal time were considered as secondary outcomes.

### Statistical Analysis

SPSS 24.0 software was used for statistical analysis. The Shapiro-Wilk test was used to determine the normality of the distribution of continuous variables. Student’s t test and Mann-Whitney test were used to analyze the normal distribution data and non-normal distribution data respectively. Categorical variables were assessed by chi-square test or Fisher’s exact test. P < 0.05 indicated a statistically significant difference.

## Results

### Clinical Characteristics and Surgical Details of Patients

In this study, a total of 89 RCC patients receiving LRN were included in the comparison (49 cases in the pre-ERAS group and 40 cases in the ERAS group). We first analyzed the demographic characteristics and clinicopathological information of the two groups (**Table 2**). The results showed that there were no significant differences between the two groups in terms of gender, age, BMI, history of smoking and alcohol, tumor side, tumor size, and pathological tissue type (P > 0.05). In terms of pathological T staging, there were 30 cases of T1, 4 cases of T2, 14 cases of T3, and 1 case of T4 in the pre-Eras group, while there were 30 cases of T1, 3 cases of T2, and 7 cases of T3 in ERAS group, with no statistically significant difference (P = 0.339). In addition, there was no statistically significant difference in operating time (124.3 ± 32.7 *vs*. 122.3 ± 33.2, P = 0.774) and estimated blood loss [60 (40-150) *vs*. 50 (40-100), P = 0.140] between the two groups.

**Table 2:** Summary of the clinical and surgical details of the patients.

Variables	Pre-ERAS	ERAS	*P* value
Age (y)	59.7 ± 7.9	57.4 ± 13.3	0.326
Sex [n (%)]			
Male	38 (77.6)	26 (65.0)	0.190
Female	11 (22.4)	14 (35.0)	
BMI (kg/m^2^)	24.5 ± 3.9	23.8 ± 3.0	0.363
Smoking history [n (%)]	12 (24.5)	8 (20.0)	0.614
Alcohol history [n (%)]	9 (18.4)	5 (12.5)	0.449
Side [n (%)]			
Right	23 (46.9)	18 (45.0)	0.855
Left	26 (53.1)	22 (55.0)	
Tumor size (cm)	5.9 ± 2.6	5.0 ± 2.2	0.071
Tumor type [n (%)]			
Clear cell carcinoma	42 (85.7)	31 (77.5)	0.315
Others	7 (14.3)	9 (22.5)	
Pathologic T stage [n (%)]			
T1	30 (61.2)	30 (75.0)	0.339
T2	4 (8.2)	3 (7.5)	
T3-T4	15 (30.6)	7 (17.5)	
Operation time (min)	124.3 ± 32.7	122.3 ± 33.2	0.774
Estimated blood loss (ml)	60 (40-150)	50 (40-100)	0.140

Values are presented as mean ± standard deviation, median (interquartile range) or n (%).

ERAS, enhanced recovery after surgery; BMI, body mass index.

### Comparison of Renal Function Parameters of Patients

Preoperative and postoperative renal function in both groups were recorded and analyzed. As shown in **Table 3**, there were no significant differences in creatinine (79.70 ± 28.28 *vs*. 77.73 ± 25.04, P = 0.732) and blood urea nitrogen (5.43 ± 1.39 *vs*. 5.76 ± 2.09, P = 0.401) between the pre-ERAS group and ERAS group before surgery. Furthermore, there were no statistically significant differences in creatinine (116.35 ± 32.71 *vs*. 115.84 ± 29.02, P = 0.940) and blood urea nitrogen (6.35 ± 1.89 *vs*. 6.71 ± 2.54, P = 0.454) after surgery between the two groups.

**Table 3:** Comparison of the patients’ renal function parameters.

Measure	Pre-ERAS	ERAS	*P* value
Creatinine (μmol/L) (preoperative)	79.70 ± 28.28	77.73 ± 25.04	0.732
Creatinine (μmol/L) (postoperative)	116.35 ± 32.71	115.84 ± 29.02	0.940
Blood Urea Nitrogen (mmol/L) (preoperative)	5.43 ± 1.39	5.76 ± 2.09	0.401
Blood Urea Nitrogen (mmol/L) (postoperative)	6.35 ± 1.89	6.71 ± 2.54	0.454

Values are presented as mean ± standard deviation.

ERAS, enhanced recovery after surgery.

### Postoperative Outcomes and Early Complications

Finally, we compared the postoperative outcomes and early complications of the two groups (**Table 4**). The total LOS and postoperative LOS in the ERAS group were significantly shortened [total LOS: 15.0 (13.5-19.5) *vs*. 12.0 (10.0-14.0), P < 0.001; postoperative LOS: 8.0 (7.0-10.0) *vs*. 7.0 (5.0-8.8), P = 0.001]. Compared with the pre-ERAS group, the hospitalization expenses of the ERAS group also decreased [51678.69 (44916.27-60242.52) *vs*. 45274.07 (39893.86-55614.36), P = 0.023]. The time of urinary catheter removal and drainage tube removal in the ERAS group were shorter than those in the pre-ERAS group [urinary catheter removal time: 2.0 (2.0-3.5) *vs*. 2.0 (1.0-2.0), P = 0.001; drainage tube removal time: 5.0 (4.0-7.0) *vs*. 4.5 (3.0-5.8), P = 0.006]. In addition, we compared the incidence of early postoperative complications between the two groups. The results showed that although there was no statistical difference, we observed fewer cases of complications in the ERAS group (P = 0.054).

**Table 4:** A comparison of the outcomes between pre-ERAS and ERAS.

Outcomes	Pre-ERAS	ERAS	*P* value
Catheter removal (d)	2.0 (2.0-3.5)	2.0 (1.0-2.0)	0.001
Drainage tube removal (d)	5.0 (4.0-7.0)	4.5 (3.0-5.8)	0.006
Total LOS (d)	15.0 (13.5-19.5)	12.0 (10.0-14.0)	<0.001
Postoperative LOS (d)	8.0 (7.0-10.0)	7.0 (5.0-8.8)	0.001
Hospitalization expenses (yuan)	51678.69 (44916.27-60242.52)	45274.07 (39893.86-55614.36)	0.023
Complications [n (%)]			
Urinary retention	1 (2.0)	0	1.000
Venous thrombosis	0	0	
Gastrointestinal discomfort	3 (6.1)	1 (2.5)	0.624
Infections	3 (6.1)	0	0.249
Postoperative hypotension	4 (8.2)	2 (5.0)	0.687
Overall complications	11 (22.4)	3 (7.5)	0.054

Values are presented as median (interquartile range) or n (%).

ERAS, enhanced recovery after surgery; LOS, length of hospital stay.

## Discussion

With the improvement of clinical diagnosis technology and the enhancement of public awareness of physical examination, more and more RCC patients have been diagnosed in time. So far, even with the introduction of immunotherapy and targeted therapy, surgical treatment is still the most effective treatment method for clinical local RCC (20–23). With the development of minimally invasive technology, LRN is currently recognized as the standard surgical procedure for the treatment of RCC. Its advantages, such as fine operation, small surgical trauma, and quick postoperative recovery, have been recognized by clinicians and patients (24). However, the surgical process inevitably destroys the integrity of the body to some extent, causing postoperative stress reaction, postoperative pain and catheter related discomfort, thus affecting the postoperative recovery of patients. ERAS’ philosophy runs through the patient’s treatment process before, during, and after surgery. By optimizing the perioperative treatment measures, the stress response of patients is reduced, the normal physiological function of the body is maintained to the maximum extent, complications are reduced, the recovery speed of patients is accelerated, and LOS is shortened (25, 26). The core of the ERAS concept is to take the patient as the center, the formulation of the plan has individual characteristics, and all measures are based on the principle of humanity.

A large number of studies have shown that ERAS protocol could relieve stress response in urological endoscopic surgery patients to varying degrees and improve postoperative rehabilitation quality and satisfaction (27–29). To date, ERAS protocol had shown significant results in radical cystectomy (30), laparoscopic partial nephrectomy (31), and laparoscopic radical prostatectomy (17), but few studies had explored its impact on LRN. In this study, we demonstrated that ERAS protocols could shorten the LOS and reduce their hospitalization expenses in patients receiving LRN.

Prolonged fasting and drinking prohibition before surgery will affect the patient’s body electrolyte balance, lead to glucose tolerance, and cause postoperative blood glucose increase, which is difficult to control. In addition, carbohydrate energy drinks intake 2 hours before surgery could keep the body in a state of energy reserve, stimulate insulin secretion, reduce insulin resistance, help maintain homeostasis, and improve patient comfort and satisfaction (32). Studies have shown that preoperative fasting for 6 hours and water deprivation for 2 hours are feasible and will not increase the risk of anesthesia aspiration (33). Preoperative mechanical intestinal preparation does more harm than good. Although a clean intestinal environment is created, the natural barrier of the intestinal tract is destroyed, resulting in imbalance of intestinal flora, disturbance of water and electrolyte balance, and prolonged postoperative intestinal recovery time (34). Positive fluid balance after surgery could lead to intestinal edema, and then cause intestinal dysfunction, and moderate fluid restriction could help patients recover intestinal function (35). In our study, early postoperative complications were assessed, and although the ERAS protocol showed a trend in favor of LRN patients, this result did not reach statistical significance. This may be due to the lower incidence of early postoperative complications and the small sample size. However, patients in the ERAS group had a shorter LOS, which could reflect a faster postoperative recovery in the ERAS group. The major limitations to the success of ERAS protocols depend on implementation capacity within medical centers and compliance with protocol guidelines. Studies have shown a lack of compliance with ERAS protocols in a multidisciplinary model of care (36). This is also a big challenge for our hospital. When implementing ERAS Protocol, we pay attention to patients’ intestinal management, fluid management and early extubation and activities, but often neglect pain management, mainly due to unclear responsibilities of pain care and patients’ wrong understanding of pain management. In this regard, we need to strengthen the management of ERAS team, clarify the division of labor among members of various disciplines, and actively strengthen health education for patients to improve patient compliance. It has been shown that with increased adherence to items across ERAS protocols, short-term outcomes are significantly improved and may also have an impact on improving long-term survival (37, 38). The steps from evidence to practice of ERAS protocols are challenging in many ways, but best practices are believed to be achievable.

Our study has some limitations: This study is a single-center retrospective study, and the results need to be further confirmed by a large-sample, multi-center study. Hospitals should pay attention to formulating their own personalized implementation plan when promoting and applying ERAS.

In summary, the ERAS protocol has significant clinical effects on RCC patients receiving LRN, which could significantly shorten LOS and reduce hospitalization expenses. It is safe and effective and is worthy of popularization and application.

## Data Availability Statement

The original contributions presented in the study are included in the article/supplementary material. Further inquiries can be directed to the corresponding author.

## Ethics Statement

The studies involving human participants were reviewed and approved by Ethics Committee of Zhongnan Hospital of Wuhan University. The patients/participants provided their written informed consent to participate in this study.

## Author Contributions

SC and XW conceived and designed the study. SC, ZH, and JS collected and sorted out patient clinical information. SC, SY, and KX analyzed and interpreted the data. SC wrote the manuscript. GW and KQ reviewed and revised the manuscript. All authors contributed to the article and approved the submitted version.

## Funding

This study was supported by the Non-profit Central Research Institute Fund of the Chinese Academy of Medical Sciences (2020-PT320-004), Improvement Project for Theranostic ability on Difficulty miscellaneous disease (Tumor) from the National Health Commission of China (ZLYNXM202006), the Fundamental Research Funds for the Central Universities (2042021kf1049 and 2042021kf1050) and Medical Science Advancement Program (Clinical Medicine) of Wuhan University (TFLC2018002).

## Conflict of Interest

The authors declare that the research was conducted in the absence of any commercial or financial relationships that could be construed as a potential conflict of interest.

## Publisher’s Note

All claims expressed in this article are solely those of the authors and do not necessarily represent those of their affiliated organizations, or those of the publisher, the editors and the reviewers. Any product that may be evaluated in this article, or claim that may be made by its manufacturer, is not guaranteed or endorsed by the publisher.
